# Cat scratch disease is an under-recognized cause of subacute meningitis

**DOI:** 10.1080/22221751.2026.2648886

**Published:** 2026-03-18

**Authors:** Michal Yakubovsky, Mirit Hershman-Sarafov, Gabriel Weber, Itzchak Levy, Dafna Yahav, Guy Choshen, Yael Paran, Avi Gadoth, Michael Giladi

**Affiliations:** a The Gray Faculty of Medical and Health Sciences, Tel Aviv University, Tel Aviv, Israel; b Department of Infectious Diseases and Infection Control, Tel Aviv Sourasky Medical Center, Tel Aviv, Israel; c Rappaport Faculty of Medicine, Technion-Israel Institute of Technology, Haifa, Israel; d Infectious Diseases and Control Unit, Bnai- Zion Medical Center, Haifa, Israel; e Infectious Diseases Unit, Carmel Medical Center, Haifa, Israel; f Infectious Diseases Unit, Sheba Medical Center, Ramat-Gan, Israel; g Encephalitis Center, Tel-Aviv Medical Center, Tel Aviv, Israel; h Neurology department, Neurology Division, Tel-Aviv Medical Center, Tel Aviv, Israel

## Introduction

Cat scratch disease (CSD) caused by *Bartonella henselae*, typically presents as self-limiting lymphadenitis; neurologic manifestations are uncommon [1]. Encephalitis is well described, but meningitis without encephalitic features is rare. While reviewing our national CSD registry, a reproducible subacute meningitis pattern was identified. We describe a case series of CSD-associated meningitis to delineate its features.

## Material and methods

A national CSD surveillance program has operated in Israel since 1991 [2]. All serologic (enzyme immunoassay) and PCR testing for *B. henselae* is performed in a single laboratory, enabling the capture of all confirmed cases [3]. Epidemiological and clinical data are obtained from physicians, medical records, and interviews and entered into a database. CSD was defined as a compatible syndrome with a confirmatory laboratory result [3], without an alternative diagnosis.

Meningitis cases were identified by the presence of symptoms and signs characteristic of meningitis (e.g. meningeal irritation, headache, or photophobia) with CSF pleocytosis. Patients with encephalitis or meningoencephalitis were excluded (*n* = 20).

Evaluation included CSF bacterial cultures and serologic testing for EBV, CMV, syphilis, Rickettsia, Brucella, and West-Nile virus (WNV). CSF PCR assays for enteroviruses, HSV, and VZV were performed per local infectious disease unit policy. Patients were excluded if any alternative diagnosis was possible.

Antimicrobials considered active against *B. henselae* included doxycycline, macrolides, and rifampin [4]. Recovery required symptom resolution and return to baseline function. A PubMed search identified previously published cases. The study was approved by the Institutional Review Board (TLV-0147-08).

## Results

Six meningitis cases were identified among 4116 CSD patients diagnosed between 1996 and 2025 (Table 1). All six were healthy young males; five owned cats. All patients had continuous or intermittent fever for a median duration of 30 days (range 7–30) before hospitalization and headache for a median of 22 days (range 5–30 days). Five patients were initially evaluated for FUO. Nuchal rigidity and photophobia occurred in two cases. Four patients had ocular involvement; three had CSD typical findings, including optic-disc-oedema, neuroretinitis, or an optic-nerve-head-lesion [5]. Lymphadenopathy, regional or visceral, occurred in three patients, and one had hepatosplenic involvement (Table 1). The course was subacute, with low-grade fever and headache, occasionally accompanied by myalgia or vomiting. Symptoms were initially mild, and most patients sought care after progression to high-grade fever and severe headache.

**Table 1.** Characteristics of 6 patients with CSD-associated subacute meningitis.

Patient	1	2	3	4	5	6
Age	34	36	30	27	37	51
Sex	male	male	male	male	male	male
Cat contact	yes	yes	yes	yes	no	yes
Lymphadenopathy	no	no	submandibular, mesentery	no	mesentery, hepatic hilum	submandibular, inguinal, mediastinal, lung hilum
Fever before admission (days)	7	30	33	30	30	14
Headache before admission (days)	7	5	30	30	30	14
Other clinical findings	photophobia, vomiting, nuchal rigidity, elevated liver enzymes	photophobia, nausea, nuchal rigidity, neuroretinitis, myalgia, tinnitus	myalgia, inflammatory optic-disc-oedema, optic-nerve-head-lesion	myalgia, bilateral knee arthralgia, vomiting, cotton-wool-spots	night sweats, weight loss, inflammatory optic-disc-oedema	hepatosplenomegaly, hepatic/splenic-lesions, vomiting
Blood on admission						
WBC (×10^3^/µL)	11	14	8	8	4	14
Neutrophils (%)	70	67	NA	73	52	80
CRP (mg/L)^a^	35	230	21	123	100	62
CSF (within 24 h)						
Opening pressure (cm H_2_O)	≤ 18	22	≤ 18	≤ 18	≤ 18	20
WBC (cell/µL)	388	43	82	11	21	10
Mononuclear cells (%)	70	67	NA	73	100	100
Glucose (mg/dL)	67	57	59	60	49	49
Protein(mg/dL)^b^	43	27	31	30	72	30
Antibiotics	doxycycline	doxycycline	azithromycin	doxycycline	doxycycline	doxycycline
Hospital stay (days)	8	7	9	9	13	8
Total illness^c^ (days)	15	50	41	39	43	22
Outcome	recovered at discharge	Recovered two weeks after discharge	recovered at discharge	recovered at discharge	recovered at discharge	recovered at discharge

Abbreviations – NA: not available; CSF: cerebrospinal fluid.

^a^ Normal value ≤5 mg/L.

^b^ Normal value 12–60 mg/dL.

^c^ Total illness duration = pre-admission symptoms + hospital stay + post-discharge recovery.

WBC count was normal, and CRP was elevated in all patients. CSF opening pressure was mostly normal with pleocytosis (median 32 cells/µL, range 10–388) and variable mononuclear predominance. CSF glucose was normal, and protein was normal/mildly elevated (Table 1).

All patients were IgM positive for *B. henselae*; five were also IgG-positive (titres 1:100 to ≥1:800). The sixth patient was initially IgM-positive/IgG-negative, showed rising IgG on repeat testing, confirming *B. henselae* infection.

CSF bacterial cultures were invariably negative. Serology for EBV, CMV, syphilis, HIV, *Rickettsia*, *Brucella,* and WNV showed no acute infection. CSF PCR for enterovirus, HSV, and VZV was performed in four patients and was negative in all.

Five patients received doxycycline and one azithromycin (median 10 days, range 7–34); none received corticosteroids (Table 1). Median hospitalization was 8.5 days (range: 7–13). All patients recovered by discharge or within two weeks thereafter. The total illness duration from symptom onset to recovery was a median of 40 days (range 15–50). No rehospitalizations were reported.

## Discussion

Meningitis is a rare and under-recognized manifestation of CSD. In both a series of 76 neurologic CSD cases and an analysis of 14,824 CSD patients, no meningitis cases were reported [1,6]. We describe six patients with meningitis without encephalitic features. All patients had headache, fever, and CSF pleocytosis, consistent with meningitis; however, the course was unexpectedly prolonged, with symptoms lasting 7–30 days before hospitalization and resolution occurring 15–50 days after onset. In contrast, adults with acute aseptic meningitis typically seek medical care within hours to days. These trajectories differ from classic acute meningitis, yet do not fully meet the criteria for chronic meningitis, which requires symptoms lasting > 4 weeks with ongoing CSF abnormalities [7]. All patients recovered. Because repeat CSF examinations were not routinely performed, we could not assess persistence of pleocytosis. Given the ambiguities in existing definitions and the absence of a clear fit with established meningitis categories, we designated these cases as subacute meningitis.

All patients in our initial cohort were male. Given the very small sample size and the inconsistent data in the literature regarding sex distribution in CSD, this likely reflects random variation. Notably, after completing the manuscript, we identified an additional female case meeting our criteria, suggesting that no sex predilection can be inferred.

A PubMed search identified six cases of CSD meningitis with sufficient clinical time-course data; five showed a subacute presentation similar to ours [8–12].

Nuchal rigidity was observed in only two patients, consistent with reports in chronic meningitis, where it is less frequent [7]. Until further data are available, we propose that *B. henselae* should be considered a potential cause of subacute or chronic meningitis, particularly when meningitis occurs with cat exposure or other CSD features.

We suspect that clinicians are unfamiliar with the atypical subacute presentation of CSD meningitis and therefore often do not request diagnostic testing, particularly serology. CSF cultures and PCR are almost always negative in CSD-related central nervous system infections [13,14]. Clinicians should look for lymphadenitis or primary lesions, which may allow PCR confirmation [14]. Extra-nodal manifestation, including visceral lymphadenopathy, neuroretinitis, optic disc oedema, or hepatosplenic CSD, all observed in our patients, should serve as important diagnostic clues. Other routine blood and CSF tests were normal or non-specific.

Given the widespread ownership of domestic cats and the growing presence of stray and feral cats in urban areas, obtaining a careful history of cat exposure is essential when evaluating aseptic meningitis. In most patients (5/6), a history of cat contact prompted testing for CSD.

CSD meningitis follows a benign course, and the benefit of antibiotics remains questionable. Although corticosteroids were not used in our cohort, they have been suggested as potentially beneficial in CSD, given the presumed immune-mediated pathogenesis [15], yet no data support their use in CSD meningitis.

The pathophysiological mechanism underlying CSD-associated meningitis remains unclear, similar to other atypical organ manifestations of Bartonella infection. Based on prior studies demonstrating intraerythrocytic bacteraemia with potential haematogenous dissemination, as well as evidence of immune-mediated phenomena, we assume that one or both of these mechanisms may contribute to CNS involvement [15].

In conclusion, we describe six cases of CSD meningitis, a rare condition with a distinctly subacute course. Cat contact, prolonged fever suggestive of FUO, and ocular, lymphadenopathic, or hepatosplenic findings should prompt evaluation for CSD. *B. henselae* should be considered a potential aetiologic agent of subacute infectious meningitis. Establishing the diagnosis is important for both patients and clinicians, as it may prevent unnecessary investigations and inappropriate treatment.

## Data Availability

Data are not publicly available, but are available from the corresponding author upon reasonable request.
